# Epithelioid cell histiocytoma with *SQSTM1-ALK* fusion: a case report

**DOI:** 10.1186/s13000-018-0704-1

**Published:** 2018-05-10

**Authors:** Ryuko Nakayama, Yuki Togashi, Satoko Baba, Yo Kaku, Yuki Teramoto, Takaki Sakurai, Hironori Haga, Kengo Takeuchi

**Affiliations:** 1 Department of Diagnostic Pathology, Kyoto University Hospital, Kyoto, Japan; 2 Pathology Project for Molecular Targets, the Cancer Institute, Japanese Foundation for Cancer Research, Tokyo, Japan; 3 Department of Dermatology, Kyoto University Hospital, Kyoto, Japan

**Keywords:** Epithelioid cell histiocytoma, Fibrous histiocytoma, *ALK* gene rearrangement, *SQSTM1-ALK* gene fusion

## Abstract

**Background:**

Epithelioid cell histiocytoma (ECH), which is also known as epithelioid benign fibrous histiocytoma, has been classified as a rare variant of fibrous histiocytoma (FH). However, the recent detection of ALK protein expression and/or *ALK* gene rearrangement in ECH suggests that it might be biologically different from conventional FH.

**Case presentation:**

A 27-year-old male presented with nodule on his left foot, which had been present for 5 years. A macroscopic examination revealed an exophytic, hyperkeratotic nodule on the dorsum of the left foot. Tumorectomy was performed, and a microscopic examination showed a subepidermal lesion composed of sheets of tumor cells with oval to round nuclei and ill-defined eosinophilic cytoplasm. The tumor cells were diffusely positive for factor XIIIa and ALK, but were negative for AE1/AE3 keratin, alpha-smooth muscle actin, CD30, CD34, CD68, PU.1, melan A, MITF, and S-100 protein. ALK immunostaining showed a diffuse cytoplasmic staining pattern. *ALK* fluorescence in situ hybridization demonstrated break-apart signals, which was suggestive of *ALK* rearrangement. A 5′-rapid amplification of cDNA ends assay detected *SQSTM1-ALK* fusion, in which exon 5 of the *SQSTM1* gene was fused to exon 20 of the *ALK* gene. The patient was free from recurrence and distant metastasis at the 1-year of follow-up.

**Conclusion:**

We were able to demonstrate the *SQSTM1-ALK* fusion gene in ECH. Practically, detecting immunopositivity for ALK and appropriate cell-lineage markers are the key to diagnosing ECH.

## Background

Epithelioid cell histiocytoma (ECH), which is also known as epithelioid benign fibrous histiocytoma, is generally considered to be an epithelioid variant of fibrous histiocytoma (FH) of the skin [1–4]. ECH is a dermal-based benign fibrohistiocytic tumor, which can mimic melanocytic, vascular, epithelial, and other histiocytic lesions. ECH usually occurs in young adults and is slightly more common in males than females [2]. ECH most commonly arises on the extremities as an erythematous dermal nodule. Although ECH is considered to be benign, cases involving multiple lesions or metastasis have also been reported [3, 5]. Histologically, this tumor is characterized by epithelioid cell proliferation in the dermis, surrounded by epidermal collarette. This pattern of epidermal changes can simulate Spitz nevus (Spitz tumor), but the tumor cells are negative for melanocytic markers and positive for dermal dendrocytic markers, such as factor XIIIa [1, 2].

Recently, anaplastic lymphoma kinase (ALK) protein expression associated with *ALK* rearrangement was reported in ECH [6–9]. However, in most of these studies the fusion partner gene was not reported. However, Jedrych et al. identified *VCL-ALK* and *SQSTM1-ALK* fusion genes in two cases of ECH [6]. More recently, two reports presented large series of gene fusion studies on ECH [10, 11]. Herein, we report a case of ECH involving *SQSTM1-ALK* gene fusion.

## Case presentation

**Fig. 1:**
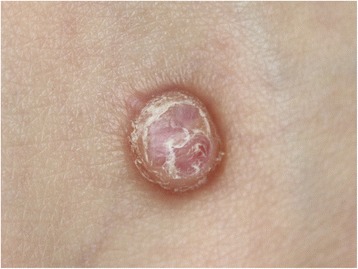
Macroscopic appearance of the ECH. The lesion presented as a 1-cm exophytic, reddish, and hyperkeratotic nodule

**Fig. 2:**
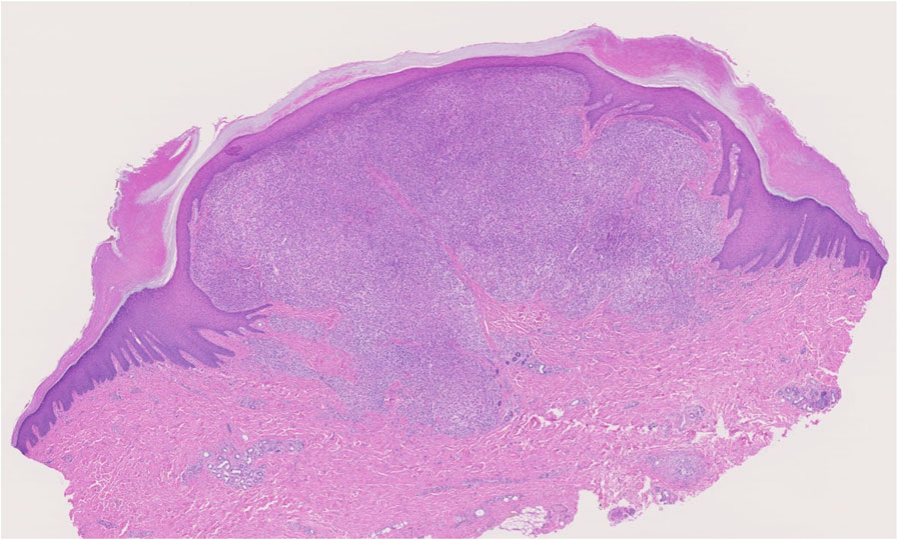
A low-power microscopic view of the lesion revealing a nodular, dermal-based tumor surrounded by an epidermal collarette

**Fig. 3:**
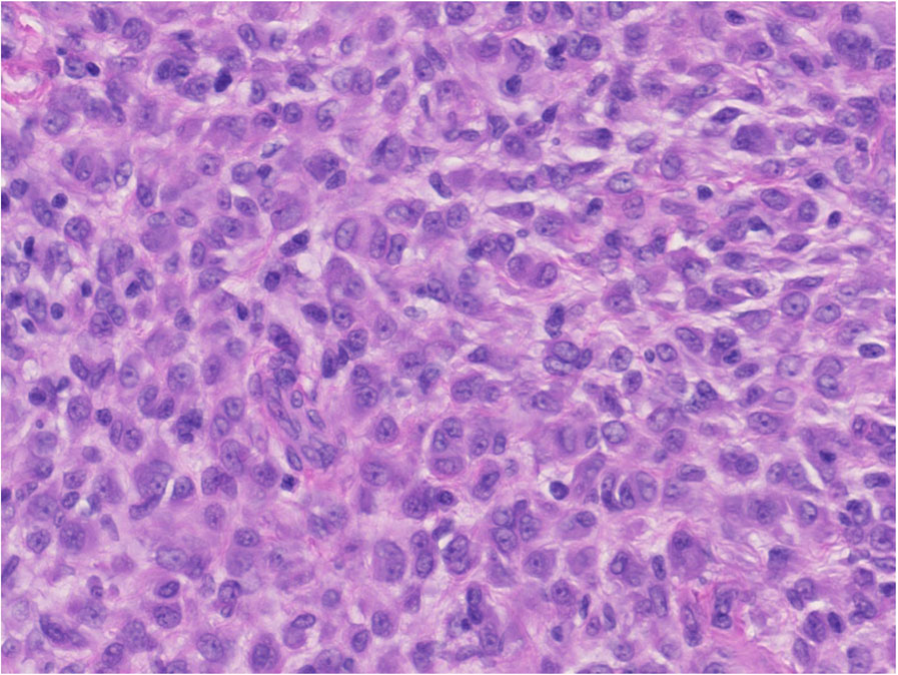
Histological findings. The tumor was composed of sheets of epithelioid cells with eosinophilic cytoplasm

**Fig. 4:**
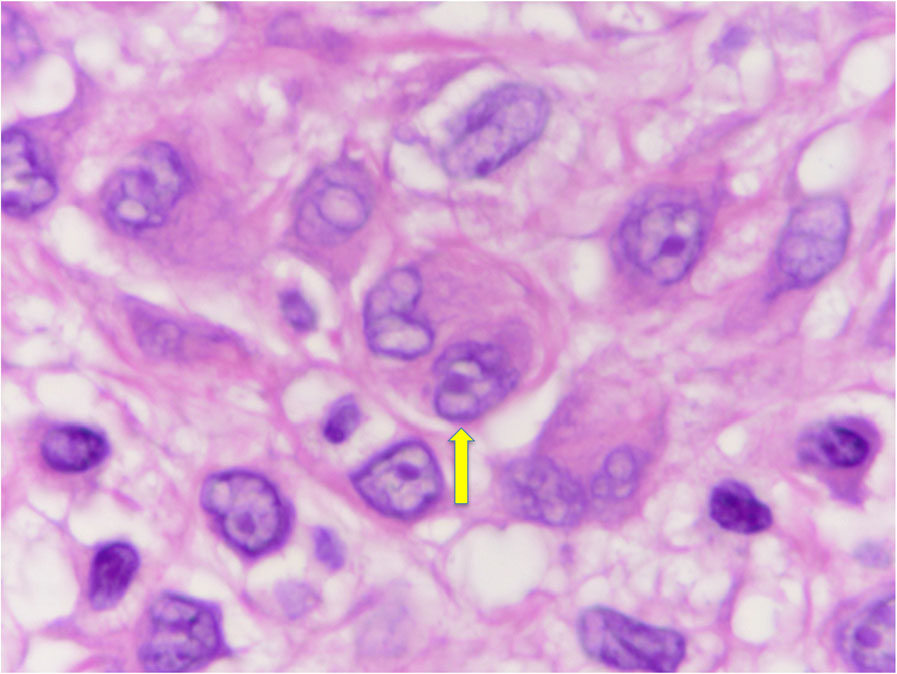
A binucleated tumor cell (arrow)

A 27-year-old male with a nodule on his left foot, which had been present for 5 years, was referred to our dermatology department. The patient stated that the nodule had grown slowly over the past few years. He was healthy, and his medical, surgical, and family history were all non-contributory. In a macroscopic examination, a 1-cm exophytic, reddish, and hyperkeratotic nodule was noted on the dorsum of the left foot (Fig. 1). The lesion was completely resected and subjected to a histological examination. A microscopic examination of hematoxylin and eosin (H&E)-stained slides performed at low magnification showed a nodular, dermal-based tumor surrounded by an epidermal collarette (Fig. 2). At higher magnification, the tumor was composed of cells with ovoid to round nuclei, small distinct nucleoli, and ill-defined eosinophilic cytoplasm (Fig. 3). Binucleated tumor cells were occasionally observed (Fig. 4). Based on the examination of the H&E-stained sections, the differential diagnoses included a cutaneous CD30-positive lymphoproliferative disorder and so-called fibrohistiocytic tumors, including conventional FH, melanoma, Spitz nevus, and ECH.

**Fig. 5:**
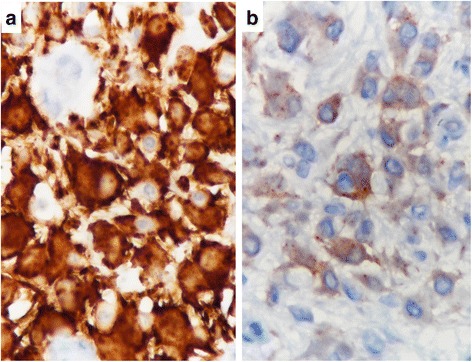
Immunohistochemistry for ALK. The immunostaining was performed using both clone 5A4 (**a**) and an anti-ALK-1 antibody (**b**) and demonstrated cytoplasmic staining

Immunohistochemically, the tumor cells showed cytoplasmic staining for factor XIIIa and ALK (the ALK staining was performed using both clone 5A4 (NICHIREI) (Fig. 5a) and an anti-ALK-1 antibody (DAKO) (Fig. 5b)), but were negative for AE1/AE3 keratin, alpha-smooth muscle actin, CD30, CD34, CD68, PU.1, melan A, MITF, and S-100 protein. The tumor’s Ki-67 labeling index was 3.5%.

**Fig. 6:**
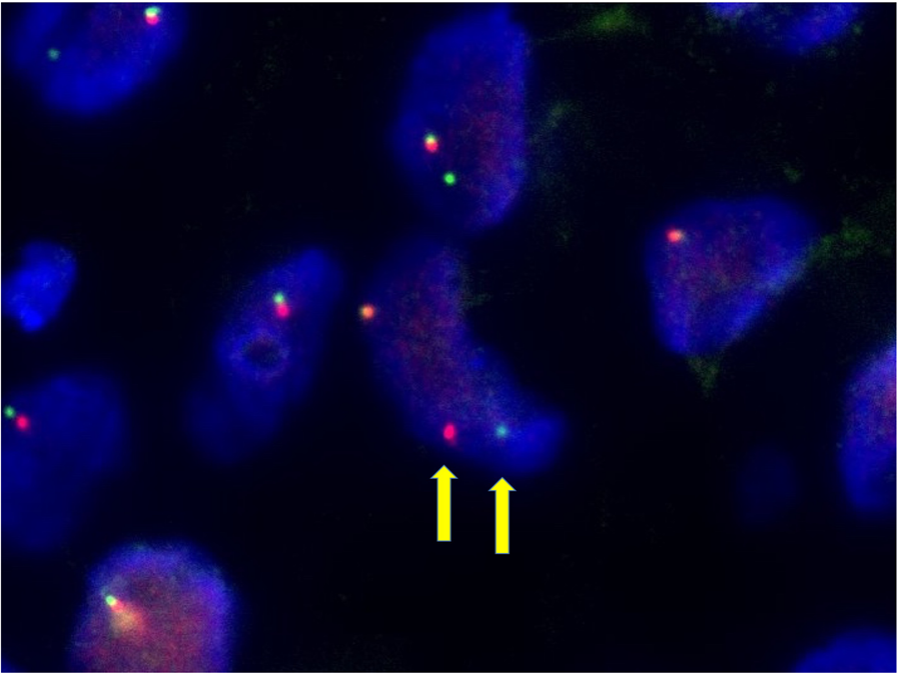
FISH analysis of *ALK* rearrangement showing split 3’*ALK* (orange) and 5’*ALK* (green) signals (arrows)

Fluorescence in situ hybridization (FISH) analysis using *ALK* break-apart probes produced a positive result, as indicated by the presence of isolated green (5’*ALK*) and orange (3’*ALK*) signals in the tumor cell nuclei, flanking the *ALK* locus at 2p23 (Fig. 6).

The histological, immunohistochemical and cytogenetic findings were compatible with ECH.

**Fig. 7:**
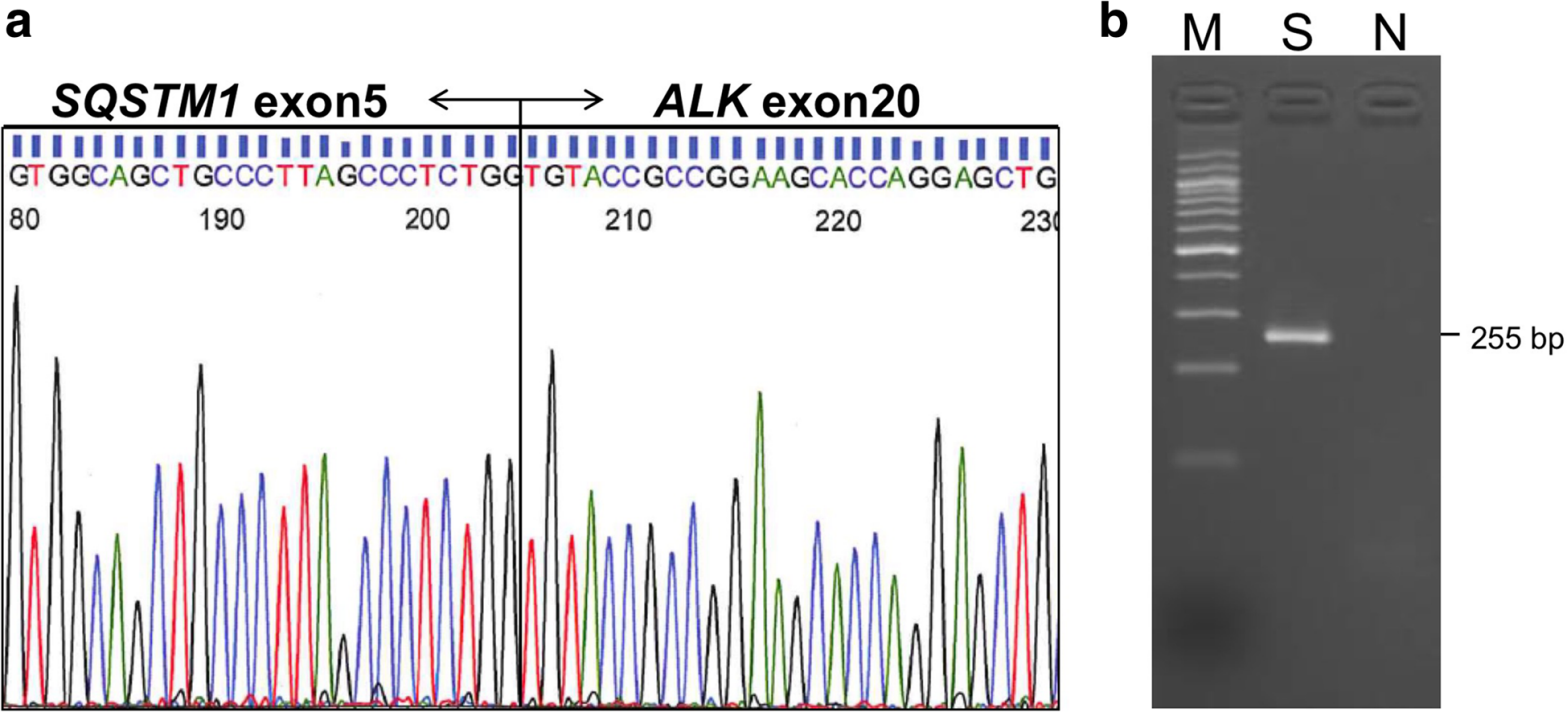
The cDNA sequence around the *SQSTM1-ALK* fusion point (**a**) and specific RT-PCR for *SQSTM1-ALK* (**b**)

**Fig. 8:**
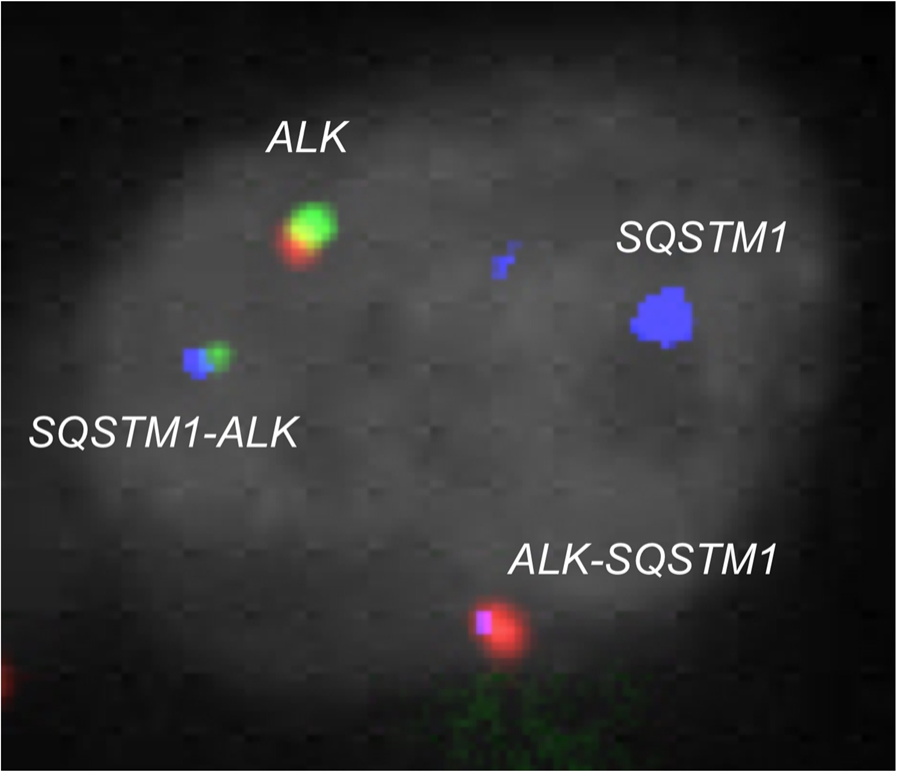
Findings of fusion FISH for *SQSTM1-ALK. 5’SQSTM1–3’ALK* (blue-green) and *5’ALK-3’SQSTM1* (red-blue) signals were detected. Note that the colors of the probe flanking the breakpoint of the *ALK* gene are opposite to those seen in Fig. 6

*SQSTM1-ALK* fusion, in which exon 5 of the *SQSTM1* gene was fused to exon 20 of the *ALK* gene, was detected with a 5′-rapid amplification of cDNA ends assay using RNA extracted from formalin-fixed paraffin-embedded tissue (Fig. 7a) [12]. To confirm the chromosome rearrangement, we performed reverse transcription polymerase chain reaction (RT-PCR) (Fig. 7b) and fusion FISH assays (Fig. 8) for *SQSTM1-ALK*. The results were also consistent with the presence of a t(2;5)(p23.1;q35.3) translocation, leading to the generation of *SQSTM1-ALK*. A final diagnosis of ECH with *SQSTM1-ALK* gene fusion was made.

The patient was well and exhibited no evidence of tumor recurrence or metastasis at the 1-year of follow-up.

## Discussion

ECH is a rare dermal neoplasm and is generally considered to be a morphological variant of FH. However, our case and recent reports suggest that ECH is actually a distinct entity, which displays a characteristic morphology, ALK immunopositivity, and *ALK* gene rearrangement. The differential diagnoses for ALK-positive tumors of the skin include ALK-positive anaplastic large cell lymphoma (ALK+ALCL) with or without systemic involvement [13] and Spitz tumors with *ALK* fusion [14]. Since the cells of both of these tumors sometimes show histiocytoid or epithelioid arrangements, immunohistochemical panels including lineage-specific markers are critical for making a diagnosis of ECH. ALCL is always positive for CD30 and some T-cell markers, such as CD3, CD4, and cytotoxic molecules, whereas Spitz tumors are positive for melanocytic markers, such as melan A, MITF, and SOX10.

After first being identified in ALCL, *ALK* has been proven to be a versatile oncogene, which contributes to a variety of tumors, including those derived from hematolymphoid, epithelial, mesenchymal, melanocytic, and neural lineages [15, 16]. Alterations in the *ALK* gene can occur through various different mechanisms, including chromosomal translocation, point mutations, and amplification. In chromosomal translocation, *ALK* fusion proteins lead to ligand-independent constitutive activation of key pathways for oncogenesis and tumor progression.

It is interesting that each type of ALK-positive skin tumor seems to harbor a different common fusion gene. The findings of Jedrych et al. [6] and our data indicate that the *SQSTM1-ALK* can be a recurrent fusion gene in ECH. The most recent large studies by two groups also suggest that *SQSTM1-ALK* is the most common fusion gene in ECH, followed by *VCL-ALK* [10, 11]*.* Other minor fusion partners include *DCTN1*, *ETV6*, *PPFIBP1*, *SPECC1L*, *TMP3*, *PRKAR2A*, *MLPH*, and *EML4* [10, 11].

The *SQSTM1* gene encodes sequestosome-1 (also known as the ubiquitin-binding protein p62), which acts as a cargo protein in selective autophagy. In addition to ECH, *SQSTM1-ALK* has also been reported in some cases of ALK-positive large B-cell lymphoma and lung cancer [12, 17]. In contrast, ALK+ALCL, a kind of T-cell lymphoma, typically involves the *NPM1-ALK* or *TPM3-ALK* fusion gene [15]. In Spitz tumors, novel *ALK* fusions, such as *CLIP1-ALK* and *GTF3C2-ALK*, have been discovered [14]. At present, the identification of a fusion gene partner of *ALK* involves a complicated process, and a combination of H&E staining and immunohistochemistry is needed to make a definitive diagnosis of ALK-positive ECH. Although staining patterns are the same, our case and a previous report showed that 5A4 clone produced stronger staining intensity than ALK1 antibody [18].

ECH and conventional FH are considered to be benign neoplasms. However, Doyle et al. [5] reported a rare case of ECH involving multiple lung metastases. The patient died of the disease after wedge resection of the lung metastasis followed by radiotherapy. Doyle et al. did not mention the results of immunohistochemistry [5], and so it is unclear whether the tumor was ALK-positive. Since other rare cases of FH involving locally aggressive growth or metastasis have been reported [19], incomplete resection should be avoided.

## Conclusions

We detected the *SQSTM1-ALK* fusion gene in a case of ECH exhibiting cytoplasmic ALK protein expression. This fusion gene might be the most common in ECH. Lineage-specific immunohistochemistry is necessary to exclude other ALK-positive skin tumors, such as ALK+ALCL of the skin or Spitz tumor with ALK fusion.

## Abbreviations

ALCL: Anaplastic large cell lymphoma
ALK: Anaplastic lymphoma kinase
ECH: Epithelioid cell histiocytoma
FH: Fibrous histiocytoma
FISH: Fluorescence in situ hybridization
